# Supplementary material for: Leveraging faith-leaders to prevent violence against women and girls: A qualitative study of evangelical faith-leaders’ perceptions in Woliso, Ethiopia
Source: PLOS Glob Public Health. 2025 Mar 26;5(3):e0003301. doi: 10.1371/journal.pgph.0003301 (PMC11942365; doi:10.1371/journal.pgph.0003301)
Supplement: S1 Appendix — (DOCX) [file pgph.0003301.s001.docx]

**Appendix 1: Faith Leader Interviews: Information sheet, consent forms, interview guide**

**Information Sheet**

We are asking you to participate in a research study titled “***Working with faith-leaders to address violence against women and girls in Ethiopia: the Case of Woliso in West Shewa***”.

**What the study is about**

The purpose of this research is to improve understanding of working with faith-leaders to address violence against women and girls in Ethiopia: the Case of Woliso in West Shew.

**Risks and discomforts**

We do not anticipate any risks from participating in this research.

**Benefits**

There are no direct benefits to participants. However, results from this research shall benefit other people now or in the future particularly in working with faith-leaders to address violence against women and girls in Ethiopia.

**Compensation for participation**

Participants will not receive any compensation.

**What will happen to the results of the study?**

The results of the study will be disseminated to the scientific, practice and policy community with a view to improve contribute to best practices and knowledge regarding the benefit and impact of working with faith-leaders to address violence against women and girls in Ethiopia.

**Audio Recording**

Audio recording devices may be used if necessary upon agreement from participants who will be transcribed subsequently.

Please sign below if you are willing to have this interview recorded. You may still participate in this study if you are not willing to have the interview recorded.

- I do not want to have this interview recorded.
- I am willing to have this interview recorded:

Signed:

Date:

**Privacy/Confidentiality**

We shall protect the participant’s privacy and confidentiality.

We will remove or code any personal information that could identify you before files are shared with other researchers to ensure that no one will be able to identify you from the information we share.

**Taking part is voluntary**

Participant's involvement is voluntary; you may refuse to participate before the study begins, discontinue at any time, or skip any questions/procedures that may make him/her feel uncomfortable

**If you have questions**

The researchers conducting this study are:-

1. Wosen Brehanu, telephone ……………………………….
2. Fitsum Zelalem, telephone………………………………….

**Consent Forms (faith-leaders)** *also available in Amharic

***Working with faith-leaders to address violence against women and girls in Ethiopia: the Case of Woliso in West Shewa***

**Consent form for Anonymised Research Participants**

**Individual Interviews**

Thank you for the opportunity to speak with you. My name is…………….., and I am a researcher. We have been commissioned by EGST to conduct a study on *Working with faith-leaders to address violence against women and girls in Ethiopia: the Case of Woliso in West Shewa.* You have been selected as one of the study informants because of your expertise and knowledge on the subject matter. With your permission, I will ask you questions about violence against women and girls.

I anticipate the interview will take approximately 1 hour. Please know that any information you provide will be completely confidential. Because I want to pay close attention during our conversation, I request your permission to use a voice recorder. We will later transcribe the recording into a word document for analysis purposes. Only the researchers in this project will have access to the transcription and recording. However, the non-personal data and the generalized findings in the form of publications from this research shall be put in publicly accessible databases and shall be available to other researchers or any other persons interested in this type of study. I want to emphasize here that the open access data cannot be traced back to you as an individual.

Your participation in this study is entirely voluntary. If you agree to participate, you can choose to stop at any time or skip any topics you do not want to answer. We ask for your support by answering questions as honestly and fully as possibly. There are no right or wrong answers; we just want to know your actual experiences and opinions.

In accordance with ethical research practice, you will keep one copy in case you want to enquire further on the study. You are free to withdraw from the interview at any time. I would now like to ask if you have any questions.

If you have any further questions regarding the research, or if you wish to make a complaint once the interview is over, please contact Dr Adamu Addissie, the principal investigator of the research (Email: adamuaddissie@gmail.com, Phone number: 0911404954)

**Consent**

I have understood the purpose of this study and how I am expected to be involved. I voluntarily accept to participate in this study and confirm my acceptance by signing/putting my thumb print below.

Participant’s name …………………………………………………………………..

Signature/Thumb print ……………………………………………………………

Date …………………..…….....................................................................................

Signature of interviewer: Date: _____/_____/________

Location of respondent:

Mode of interview: (e.g. face to face, telephone, zoom)

**Interview guide – faith-leaders**

Hello, [interviewee’s name]. We really appreciate your involvement with the Channels of Hope workshops and for you taking the time to be here with us today for the interview. My name is [your name] and I am a [role] within this intervention/research project.

As you are aware, the study aims to improve gender equality and address intimate partner violence through working with faith-leaders, like yourself, through the Channels of Hope methodology. The main research question that we seek to answer is What are the perceptions and experience of faith-leaders, trainers and the community on the implementation process of the faith-leader training on gender and IPV? To determine the potential for further application of this process, we need to evaluate your experiences and how you felt that transformative learning happens. That is why we have asked you to be here today to participate in the interview. Do you have any additional questions, comments, or concerns before we get started with the interview?

Questions:

Establish a common ground

2) What do you consider as the role of women and girls in society or your community?

a. How do you view women and girls in relation to men?

b. What are your views on using beating on women?

c. Do you think that women’s level of autonomy in your community is too limited, just right, or too great?

2) What are your concerns and challenges regarding women and girls in your communities/congregation?

a. Do you think violence against women is an issue in your community?

b. What types of violence? Why is it happening?

3) What is your interpretation of the Bible’s teachings and practice on the role of women in society?

3) What are your hopes and solutions for women and girls in your communities/congregation?

4) What can you as a faith-leader do to address some of the concerns you mentioned?

5) What are your expectations of the Channels of Hope workshops and process?
