# Supplementary material for: Leveraging faith-leaders to prevent violence against women and girls: A qualitative study of evangelical faith-leaders’ perceptions in Woliso, Ethiopia
Source: PLOS Glob Public Health. 2025 Mar 26;5(3):e0003301. doi: 10.1371/journal.pgph.0003301 (PMC11942365; doi:10.1371/journal.pgph.0003301)
Supplement: S2 Appendix — (DOCX) [file pgph.0003301.s002.docx]

Ethiopian Graduate School of Theology

CoH for Gender CHAT Workshop Schedule at Weliso

**Objectives of the workshop: at the end of the 3-day workshop Nov 14- 18, 2022, the participants shall have:**

1. Have gained a better understanding of gender relationships from a faith and interdisciplinary perspectives
2. Have explored norms, attitudes, and value systems informed by culture and theology that tend to reinforce inequality and lack of equity;
3. Have recognized common justifications for Gender Based Violence , and equipped to frame appropriate responses;
4. Be able to prepare and deliver Channels of Hope for Gender content and methodology to achieve change of attitudes and knowledge increase about faith perspectives on gender
5. Have caught a vision for transformed gender relationships for their families, faith communities and the communities at large

| **Time and Date** | **Day 1** | **Facilitator** | **Day 2** | **Facilitator** | **Day 3** | **Facilitator** |
| --- | --- | --- | --- | --- | --- | --- |
| **2:30 - 9:00 am** | Registration | Henok | Recap & Orientation for the day | Mengistu | Recap & orientation for the day | Wosen |
| **9:00 - 10:45 am** | Introduction & Climate setting - CoH Objectives - Overview of Workshop Themes | Mengistu | Type and consequences of GVB | Wosen | Leadership – Gender based or gift based? | Mengistu |
| **10:45 - 11:00 am** | **Tea Break** |  | **Tea Break** |  | **Tea Break** |  |
| **11:00 - 12:30 am** | Who I am (as male/female) in our community | Wosen | Biblical and Theological foundation  Harmony in Gender relation | Mengistu | The response of church towards Gender equality and GBV | Wosen |
|  | My most memorable childhood experiences as a boy/ girl Agree/disagree | Mengistu |  |  |  |  |
| **12:30- 1:30 PM** | **LUNCH** |  | **LUNCH** |  | **LUNCH** |  |
| **1:30 - 3:30 PM** | Gender & Sex? Gender Box | Wosen | Gender relations in Disharmony | Wosen | Wrap up, Reflection & Close for the Day | Mengistu |
|  | What influences our worldview? Circle of influence |  |  |  |  |  |
| **3:30 - 3:45 PM** | **Tea Break** |  | **Tea Break** |  | **Tea Break** |  |
| **3:45 - 4: 45 PM** | Gender based violence | Mengistu | Harmony made possible | Mengistu |  |  |
|  | Wrap Up/ Close for the Day | Wosen | Wrap Up/ Close for the Day | Wosen |  |  |
