# Supplementary material for: Leveraging faith-leaders to prevent violence against women and girls: A qualitative study of evangelical faith-leaders’ perceptions in Woliso, Ethiopia
Source: PLOS Glob Public Health. 2025 Mar 26;5(3):e0003301. doi: 10.1371/journal.pgph.0003301 (PMC11942365; doi:10.1371/journal.pgph.0003301)
Supplement: Attachment — Submitted filename: PLOS Global Public Health Reviewer Response.docx [file pgph.0003301.s006.docx]

PLOS Global Public Health

**Leveraging Faith-Leaders to Prevent Violence against Women and Girls: A Qualitative Study of Evangelical Faith-leaders’ Perceptions in Woliso, Ethiopia**

Reviewer Response

| **Journal Requirements** | **Response** |
| --- | --- |
| Please include a complete copy of PLOS’ questionnaire on inclusivity in global research in your revised manuscript. Our policy for research in this area aims to improve transparency in the reporting of research performed outside of researchers’ own country or community. The policy applies to researchers who have travelled to a different country to conduct research, research with Indigenous populations or their lands, and research on cultural artefacts. The questionnaire can also be requested at the journal’s discretion for any other submissions, even if these conditions are not met.  Please find more information on the policy and a link to download a blank copy of the questionnaire here: <https://journals.plos.org/globalpublichealth/s/best-practices-in-research-reporting>. Please upload a completed version of your questionnaire as Supporting Information when you resubmit your manuscript. | The questionnaire has been completed and uploaded as a supplementary document, per the guidelines. Information regarding inclusivity in global research has been added to the text in Methods on lines 285-290. |
| **Reviewer #1** |  |
| This paper began as an interesting look at the role of faith leaders in changing violence against women and girls in their faith communities in a context where violence is rampant. However, the methodology described was very confusing, which led me to quickly lose enthusiasm for the paper. Furthermore, it is unclear what interventions would look like to raise awareness and promote transformational change on VAWG in this context. I am still not sure if the training was the intervention, or if the point of this research was to determine what such an intervention would look like. This paper needs major revisions starting with the methodology in order to be suitable for publication. | We appreciate your feedback and taking the time to provide insightful comments. We agree that the methods section was confusing and not sufficiently clear. This is part of a larger project and we have made that link clearer by making major revisions to the Methods section (marked in red on the document on lines 172 – 242). This also allowed us to clarify the specific aim of the paper, which we agree needed clarifying (Lines 167-169). Specific comments and changes have been addressed and elaborated on below. |
| "Equality” and “equity” are used in the background interchangeably. Need consistency, and to ensure the correct term is being used. | Thank you for pointing this out as definitional clarity is very important with this type of study. Among the team we have decided to use equality more readily throughout the paper, and have provided a definition of both equality and equity on line 103-108. |
| Bottom of page 5: have interventions for males in religious settings been introduced in Ethiopia? | Yes, there has been a program called Project dldl that has focused on Ethiopian Orthodox faith leaders. We have now included a section on this on lines 159 -162. |
| Is part of the study aim to understand how VAWG could be addressed by faith leaders, or what types of interventions would be feasible and acceptable? That seems to be missing from the study objectives but would seemingly be one of the most important pieces. | Yes, we agree that wasn’t clear. The aim was to understand what role faith-leaders felt they could play in addressing VAWG and what part they could play. This is now added and clarified in the aim (lines 167-169) and throughout the methods section. |
| CoHG seems to be focused on transforming more than knowledge; attitudes, norms, and behaviors of faith communities as well? | Yes, that is correct it is addressing more than just knowledge, but this is the terminology used by the World of Vision organization and is consistent with similar literature. As part of our revisions on the CoHG this section has been streamlined, as the focus of this study is on the perceptions of the faith-leaders. Instead we provide some more information on the format and material of the workshops on lines 220 – 228 and with additional material now provided in the appendix. |
| Line 177: Should be “A later study stage involved conducting interviews…” Was the stage included in the current research being reported? | No, this later stage was not included in this research being reported. This study has been linked to the wider project in the methods section. We have added information specifying the three different project studies on lines 181-188. |
| It was unclear to me whether this was an evaluation of the intervention or formative research to create or adapt an intervention. I believe it is the latter, but the study objectives and methodology should be revised to make this abundantly clear. Was the observation of the training? I thought the interviews were to inform the training? | We agree that this became unclear based on lack of clarity in the methods section. This study is formative research on faith-leaders' perspectives on VAWG and the role they think they could play. That knowledge was then used in a later study to inform community-based interventions. This has now been explained more explicitly in the methods section (see comments above). Additionally, we now refer to the workshops as Workshop #1 and Workshop #2 in the text rather than using the names Envision and CHAT to improve the reader understandability of the methods/results. |
| What was included in the observation? How was it done? What was noted? There are no details on this element. | Agreed that this was too thin, now added with a separate section within the methods section (lines 215-228). We have also included the workshop agenda and relevant sections from the training guide to the appendix. |
| How many people did the coding? How was coding reliability assessed. | We appreciate you pointing this out as it is not fully explained in our methods section. The coding was done initially by two team members and then it was double checked with other team members, and theme generation was done through a collaborative and iterative process of team discussion and refining. This is now elaborated in the text in the Data Analysis section (lines 230-242). |
| Line 235: “This was mitigated with consistent team meetings.” What sort of issues were discussed or create a point of contention among the team members? Also, what is “good research practice” for methodological reflexivity? | This included discussions about our own values and norms. Whilst we were on the same page with most discussions there were some where men and women in the team had slightly differing views. For example, with regards to financial control being a form of violence. We wish to not describe this level of detail in this section.  For the good research practice, that refers to the guidelines from the Swedish Research Council. This information has been added in lines 270-271. |
| It seems the results are the themes that came out of the interviews. Where are the results of the observations? Perhaps this is an extension of the confusion around the methods. It is unclear which data are from observed training sessions and which are from individual interviews.  It also does not seem that participants were asked specifically what role they see them having in changing the perceptions of their faith communities regarding violence against women and girls. They were willing to talk about how faith and culture are intertwined in a way that perpetuates violence, but it is not clear how they think this will change. | The results are the themes that came out of both the results and workshop observations. We coded this data together. Described more clearly in the methods section. We have added a sentence in the beginning of the results at line 293 – 295 to clarify. Also, hopefully the clarifications made above the methods section have helped to improve the understanding of the results. Each of the interviews is also labeled with the interview or workshop that it is from. We added the term workshop#1 or workshop #2 to further improve clarity.  In terms of the role of the faith-leaders, they were asked this question about how they see their role. We provided additional information on the topics included in the interview guide on lines 210 – 212. Moreover, there were activities in the workshop where faith-leaders discussed what they could do in their community. We did not evaluate perceptions on their interventions in the community in this study, this is a separate study. This has also been described more clearly in the methods section. |
| Again, it is not clear how the faith leaders would fulfill their role as change agents. They are interested in engaging on the topic of violence, but what does this research show they will need to do in order to affect change in their faith communities? | We describe the whole project and the fact that this is a formative study more clearly in the methods section. As mentioned, we agree that this was confusing. Identifying faith-leaders’ perspectives on VAWG and how they see their role in addressing VAWG (in other words the findings from this study) helped tailor future trainings for faith-leaders which is described in study 2. Additionally, we have revised the discussion section on the potential impact of faith-leaders as change agents to more explicitly link with the data presented and the wider existing research on faith-leaders in VAWG prevention. |
| Was the efficacy of the training reported? I missed it. Again, there is confusion around the methodology and what exactly is being assessed here. Clarity is needed. | Thank you for pointing this out because no, the effectiveness of the intervention is not being reported here in this study. This is described more clearly in the methods section (refer to comments above). Rather this is a formative study that explores faith-leaders views and their perspectives of their role in addressing VAWG. We hope that the changes to the methodology section notes above help to resolve this confusion. |
| Was Data analysis only conducted by one person? They may be more objective if not part of the data collection but how did you assess reliability? | (Response the same as the question about coding above): We appreciate you pointing this out as it is not fully explained in our methods section. The coding was done initially by two team members and then it was double checked with other team members, and theme generation was done through a collaborative iterative process of team discussion and refining. This is now elaborated in the text in the Data Analysis section (lines 230-242). |
| Another limitation is not knowing the baseline attitudes towards violence of the faith leader participants. | Considering the methodological confusion earlier on then this would be a limitation. However now that we have clarified the methodology above, we hope that this clears this up. The interviews were the way to get baseline information on attitudes, and many of the workshop discussion observations were grounded in their baseline beliefs. Thus, we believe that this cannot be a study limitation. |
| Editing is required throughout the manuscript for grammatical errors. | Thank you for noting this. We have now had two separate native-English speaking team members read through the text thoroughly for grammatical errors. |
| **Reviewer #2** |  |
| There are some problems with terminology, especially in the Introduction, in terms of the way that sex and gender are used interchangeably. “Male dominated views” are better termed “patriarchal views”, and “social and cultural factors associated with being male or female” should read “(…) associated with being men and women. I would urge the authors to be more careful about using the correct gendered (as opposed to sexed) language throughout the manuscript. | Thank you for pointing out these challenges in terminology. We had decided to use the term male dominated rather than patriarchal because in the Ethiopian context the term Patriarchy typically refers to the hierarchy within the Orthodox Church specifically. It therefore does not actually have a negative connotation in Ethiopia but rather one of respect and devotion.  As we hope that this research will be understood in both an Ethiopian and an international context, we have switched to using ‘patriarchal’ but have provided a clear definition of this in the introduction on lines 92-93 to clarify in the Ethiopian context.  For the gendered/sexed terms, thank you for pointing this out at it is a critical component of the research. The choice to use male or female was initial taken because some of the collaborators in Ethiopia were not comfortable the more Western conception of gender since this is often associated with LGBTQI concepts which is a very sensitive topic. We have now clarified this in the introduction on lines 102-105 and adjusted throughout the text to be man or woman as you can see via track changes. |
| Likewise, “tradition” is a complex theoretical construct that is taken as naked fact (e.g. “traditional masculinity”). What does this mean, practically-speaking? | It is important to note this, and we have elaborated with a definition of traditional masculinity that we will refer to throughout the text on lines 87-101. It is a term that is used also throughout the interview and workshop observational data by the faith-leaders. In this instance we cannot know explicitly what they meant by interpreted it based on our own experiences and the contextual clues from the data. |
| Page 3: “a critical factor is religion”. A critical factor in what? | We appreciate you noting that this thought is incomplete. We have elaborated that religion is a critical factor in gender norms and VAWG. See track changes on lines 110-112. |
| P. 3: “believe in other realities.” What does this mean? | Thank you for bringing this up, as the definition of religion is important within our study. This ‘belief in other realities’ comes from Schilbrak’s much larger discussion of attempting to define the complex topic of religion. We do understand that this term is vague, but that allows for a wide encompassing of different religions including monotheism, polytheism, and non-theistic religions. For the sake of being succinct, we have decided to leave this as is in the text and allow the reader to refer to the cited text for a more detailed explanation. |
| There are some methodological strengths to this paper (the reflexivity section; analytic approach). I would like a bit more information, however. You state that this is a qualitative study. Any particular kind? A generic qualitative study? An ethnographic study? This should be explained and referenced. Also, could you explain more about the sense in which it is a participatory action intervention study? | We appreciate your kind comment about the strengths of the methodology. Additionally, it is useful to provide more information on methodology to provide clarity to the reader. The study was a generic qualitative study with a constructionist perspective, which we have now clarified on lines 174. 188. |
| It would be helpful to summarize under the Data Source and Collection heading the kinds of topics that were explored in the interview guide. Also, who exactly carried out the interviews. I gather that the interviewers were not involved in the analysis. Why not? | Thank you for the suggestion, we have now added a sentence with the topics of the interview guide (line 233-23) in addition to having it as a supplementary file. We have also added the initials of those involved in the interviews/data collection. These team members were also involved in the iterative process of the theme generation and we have clarified this on lines 253 – 255. |
| The reflexivity section is great, however you state that “the team followed current good research practice [in terms of methodological reflexivity]”. Which is please explain this explicitly. | Yes, it is good to note that this was not fully clear. The good research practice is a series of guidelines from The Swedish Research Council, which we have now cited on lines 280-281. |
| The material could be better situated within the broader context of religion in Ethiopia, especially given the authors note that religion, culture, and broader society are inseparable. While this study was carried out with Evangelical Christians, most Ethiopian Christians are Ethiopian Orthodox Christians, and Ethiopia also has a large Muslim population. Relatedly, I notice that there seems to be some “othering” going on among your participants (e.g., the participant who states that it is “unbelievers” who sometimes have problems with VAG; that its especially women in rural areas that housemaids who struggle with this). This seems important, as it pushes the issue onto others, potentially allowing study participants to avoid responsibility to address these issues in their own families, communities, congregations etc. This seems to be an important thread that is not taken it. | We have added some more information on the importance of Evangelism in the introduction on lines 163-166. However, we have decided not to go farther into depth on the other religious groups in this paper as this is being done elsewhere.    Your point on “othering” is interesting and we will reflect further on this for our other studies. We have clarified, in that section, that the reference to housemaids was described as being a part of an issue in their communities. Upon reflection, the team felt that the point on the nonbelievers was a bit distracting and also somewhat unclear, we have decided to take that out of the quote on lines 331 -334. We then address blame shifting a bit in the discussion on lines 537 -549 in discussing the interrelated nature of social and religious norms. |
| p. 14: a word is missing in the final sentence (blaming women who fail to dress in traditional for rape). | Thank you for pointing out this typo. We have now addressed by shifting the sentence syntax on lines 380-382. We have also read through the text for other grammatical errors. |
| Another topic that is raised in the results but is missing from the analysis is the ways in which responsibility for limiting VAG is being placed on women, both by men and women. For example, the issue of women not dressing modestly leading to rape, and also the woman on page 15 who encourages women with unfaithful husbands to consider what they themselves could do differently (the implication here being that he is looking elsewhere because he is unsatisfied at home). I think this is an important analytic piece that could be pursued (this is merely a suggestion for how the piece could be strengthened; I do not consider it imperative). | We agree that this is an interesting concept to tease out of the data. We have added a few sentences to address this idea within the definitional clarity section on lines 521-524. |
| I am confused by the first quote under the “Personal Responsibility” heading. Could you please explain more clearly what this quote means, and how it relates to personal responsibility? | With a quote isolated from the rest of the text it can be confusing, so thank you for requesting clarification. We have now added two sentences of further clarification of the quote at lines 463 – 465. Additionally, we have renamed the theme itself to ‘Individual Responsibility’ |
| Also, I wonder whether you could engage with a discussion of whether scripture itself is contradictory (as a secular agnostic, Christian scripture certainly seems to be contradictory to me), and what the implications of this are for interventions that mobilize scripture to combat GBV. How do people reconcile contradiction? How do you decide which sections are relevant, and which interpretations are correct? | This is a great point and something we have actually talked about a lot in the team. We agree with the contradictory elements and much of theology engages with this in detail that we feel is beyond the scope of this paper. In our reflexivity section, we describe that we believe an interpretation of scriptures that denounces VAWG to be true and this is also the approach of COH training. We have also added a sentence on lines 498-500 to describe how this inherent contradictory nature of the scripture can impact the heterogeneity in faith-leader perspectives. |
| The discussion of Definitional Clarity is good. | Thank you! |
| The Limitations section: it seems contradictory to state that an outside analyst is somehow more objective, when you have stated (correctly) in the methods section that objectivity is impossible given everyone is situated vis a vis a research context in which the meanings are themselves contested and socially constructed. I would perhaps rephrase this to explain that a diversity of analytic perspectives enhances the trustworthiness of the data. | This is a critically important comment, so thank you for pointing it out. As a qualitative study especially everyone is coming from different perspectives and bias which are innately included within the research. We have adjusted this sentence on line 615-619. |

Note: line numbers refer to information in the track changes version of the manuscript.
