# Supplementary material for: Leveraging faith-leaders to prevent violence against women and girls: A qualitative study of evangelical faith-leaders’ perceptions in Woliso, Ethiopia
Source: PLOS Glob Public Health. 2025 Mar 26;5(3):e0003301. doi: 10.1371/journal.pgph.0003301 (PMC11942365; doi:10.1371/journal.pgph.0003301)
Supplement: Attachment — Submitted filename: Response to Reviewer_VAWG #1_PLOS GH (2).docx [file pgph.0003301.s007.docx]

Reviewer Rebuttal Letter

| **Journal Requirements** | |
| --- | --- |
| Please review your reference list to ensure that it is complete and correct. If you have cited papers that have been retracted, please include the rationale for doing so in the manuscript text, or remove these references and replace them with relevant current references. Any changes to the reference list should be mentioned in the rebuttal letter that accompanies your revised manuscript. If you need to cite a retracted article, indicate the article’s retracted status in the References list and also include a citation and full reference for the retraction notice. | In regard to the reference list we have double checked all of the references and made some slight adjustments as listed below;   - Levant et al. (2013), to elaborate on the term of avoiding femininity per a reviewer comment, - Cited the follow-up study to this manuscript, which has been recently published (Gobbo, et al (2024)), - Updated the link on Schilbrack (2013) - The Tomkins, et al. (2015) paper had an edit to one sentence that was noted and adjusted for in the online version of the article in the Lancet. Thus, the version that has been cited is the corrected version. <https://www.thelancet.com/journals/lancet/article/PIIS0140-6736(15)00699-6/fulltext> We determined that this meant the article was acceptable for inclusion as a source in this manuscript. - Fixed a technical error with Wolff, et al (2008) to have the correct title in the reference list. - Adjusted title on Palm & Le Roux (2017) |
| **Additional Editor Comments** | |
| Ensure that sex and gender are not used interchangeably | We have now gone back through the text and adjust to a more consistent and correct usage of sex and gender where appropriate. In the results, there remains some conflation but as that is the perspectives of the participants, we have maintained their usage. (Further response in comment to Reviewer #2) |
| Provide justification on combining various forms of qualitative data versus analyzing them separately. | To describe the rationale for the use of combined data forms, we have added a few sentences on lines 199-204 at the beginning of the Data Source and Collection section. |
| **Response to Questions (No)** | |
| 5. Is the manuscript presented in an intelligible fashion and written in standard English?  PLOS Global Public Health does not copyedit accepted manuscripts, so the language in submitted articles must be clear, correct, and unambiguous. Any typographical or grammatical errors should be corrected at revision, so please note any specific errors here.  Reviewer #2: (No Response)  Reviewer #3: No | Thank you for noting some syntax and grammatical errors. We have now had two native English speakers read through the text again for clarification. Additionally, we have put the paper through Grammarly to check for any further typographical errors. |
| **Reviewer #2** | |
| This paper is much improved from its original version, and I now have only minor suggestions/concerns. The methods section is now very good. My minor concerns are as follows:  My main concern is that there is still inappropriate conflation of gender and sex (in the sense that they are used interchangeably in situations where there is inaccurate). Yes, I understand that the distinction is problematic/ not meaningful for many study participants, and thus in the results we would expect these concepts to be conflated. But in descriptive and/or analytic sections of the paper, using them interchangeably is frankly inaccurate. For instance, on page 3-4, where it reads "in this study gender equality refers to the notion that men and women have the same access to opportunities and life changes and that they are not dependent on or constrained by their sex" should really use consistent GENDER as opposed to sex language throughout. Likewise, the table on page 12 lists the "gender" of participants as being male and female, when it should be written as "men" and "women." Male and female quite simply is not their gender, so the table is inaccurate in its current form. | Thank you for pointing out some continued conflation of sex and gender. This is a critically important consideration for a study in this field, and complex due to the study context. The team did have extensive conversations around the use of gender and sex, as the use of the terms is complex within the study setting. There are some stigmas and connotations around the idea of gender. From your comments we have decided that it does make sense to more consistently use gender-based terminology, rather than sex. We have gone through the text and adjusted for any remaining issues we found and for the specific comments you raised. |
| p. 3: in the first paragraph of the paper you state that "VAWG is defined as "....". Defined by whom? Surely there is more than one definition, and I think it makes sense to specify whose definition you are using. | Thank you for noting this. We have now clarified that the definition is from the World Health Organization on line 79. |
| p. 3: "avoidance of femininity" is a bit vague. Surely traditional masculinity can entail interacting (in specific ways) with normatively feminine women. I think this could be worded to be more clear. | The concept of ‘avoidance of femininity’ comes from the development of a Masculinity scale from Levant, et al. (2013). It was then also used in an updated study examining the perceptions of traditional masculinity from Iacoviello, et al (2021). In the scale, ‘Avoidance of Femininity’ refers to men watching football instead of soap operas, preference for action movies over romance novels, and boys preferring to play with trucks rather than dolls. Based on this concept and to clarify, we have adjusted the phrase to say “avoidance of participating in feminine activities” on line 96. |
| p. 5: "leveraging re-interpretations." Re-interpretations of what, exactly? | To further clarify this line, we have added a clause “of religious texts” to the sentence on line 131. |
| p. 14 the sentence "additionally, the analysis held indications of some faith-leaders understanding economic, social, or geographic differences" is unclear to me. | We have adapted the sentence to improve clarify of meaning on lines 323 – 326. |
| p. 20: the quote which states "he loved the Church by undermining himself" is unclear, I think because "undermining" is probably not being used the way it is typically used in English. Could you clarify, perhaps in a square bracket, what is meant here? | We appreciate you noting this confusion as it is a translation gap from the original Amharic. We have now added “humbling” in brackets next to undermining to try and clarify the intended meaning rather than the direct translation. |
| p. 26: forth should read "fourth" | Thank you for pointing this out. Along with other grammatical changes this has been adjusted on line 589. |
| **Reviewer #3** | |
| There were minor grammatical errors and a need for rephrasing, specifically on lines 93 and 140. Authors should do another thorough review for minor mistakes. | We appreciate your editing feedback. We have now had two native English speakers read through again for clarity and put the manuscript through Grammarly to check for any remaining typographical errors. We have specifically addressed the errors you pointed out in lines 93 and 140. Both these sentences have been reworded. |
| The analysis is sound, and the authors clearly outline how it took place. However, there are two sources of qualitative data: (1) interview transcripts and (2) workshop notes, which for some would be considered a mixed qualitative analysis because of the mode of data collection. It would strengthen the methods section if the authors described why they chose to combine the interview data and workshop data rather than conducting separate analyses. Questions I would recommend answering: How does the combination of the data strengthen the analysis and results? What are the limitations of combining the data? | Yes, we again that the methods section could be further strengthened by adding the rationale for using the two sources of data. Lines 200 – 204 in the Data Source and Collection section now describe that we used the two sources in order to triangulate and increase data reliability. We elaborate on the benefits of each type of data together. In the limitations section of the discussion we now provide a sentence stating that this may also be a limitation for reducing comparability. The changes are made on lines 599 – 601. |
| The discussion section offers significant insights into the field, particularly the use of faith leaders in combating GBV through social-behavioral interventions. This is a commendable contribution to the field. | Thank you for this and we appreciate your recognition of the works contribution to the field. |
