# Supplementary material for: Leveraging faith-leaders to prevent violence against women and girls: A qualitative study of evangelical faith-leaders’ perceptions in Woliso, Ethiopia
Source: PLOS Glob Public Health. 2025 Mar 26;5(3):e0003301. doi: 10.1371/journal.pgph.0003301 (PMC11942365; doi:10.1371/journal.pgph.0003301)
Supplement: Attachment — Submitted filename: PLOS Global Health Reviewer Response.docx [file pgph.0003301.s008.docx]

PLOS Global Health

Reviewer Response –

Dec 2024

I recommend using a language editing service to identify and correct a few remaining spelling and grammatical errors. For example:

-Using one of UK or US English consistently throughout the text

- Thank you for the very focused comments and grammatical suggestions. We have now used US English consistently throughout. We put the text through Grammarly and adjusted using track changes with the assistance of the software and based on your comments.

-Page 8, line 200: Should be “Hawthorne effect”

-Page 9, line 217: Abbreviations should be written in brackets after the first time a term is used

- Page 9, line 231: Should be “in Nvivo”

-Page 10, line 258: There should be an apostrophe at the end of “wives”

-Page 11, line 263: “that” is written twice

-Table 1: Should be “gender”

-Page 17, line 382: Should be “religious and social norms”

-Page 19, line 429: Should be “gender unequal”

-Page 25, line 566: There should be an “a” before the word useful

-Page 26, line 586: There should be an apostrophe in the word “participants”

The following changes are required for acceptance:

1. Please use the COREQ checklist, or another relevant reporting checklist, to ensure complete reporting of the methods and results. A supplementary document should be included detailing where in the manuscript the checklist items are reported.
   a. We have now added a supplementary file (Additional File 5), and made some slight adjustments throughout the text to ensure accurate reporting.
2. The terms (in)equity and (in)equality are still used interchangeably in some parts of the manuscript. Please review these sections and revise accordingly (page 6, lines 144-146; page 18, lines 403-406 where title mentions equality but then paragraph refers to equity;)
   a. Thank you for pointing out these inconsistencies. We have now gone back through the text and ensured that the only time equity is discussed is in the definition at the beginning and the rest of the text references equality.
3. Please provide additional information to clarify the following points:

- Page 6, line 155: It's not clear what the percentages are referring to. Are you indicating that 13 to 30% of all women in the regions participated in the program?
  - In order to clarify the statement we have instead mentioned the number of women trained who then guided the programs.
- Page 7, line 187: Please report the rate of VAWG in Oromia
  - We have now reported that 38% of women in Oromia reported physical, sexual, or emotional abuse.
- Page 9, line 220: Please clarify what is meant by “high-level leader” as those not familiar with Evangelical church system in Ethiopia will not know what this means
  - To help to clarify the hierarchy structure in the Church, we have added a few sentences under the Study participants section on lines 194-202. Then on pg. 9, line 229-230, we have changed the wording to top-level leaders to be consistent with the description above.
- Page 9, lines 226-227: It is indicated that the workshops were not audio-recorded but no information is provided about interviews. Please specify whether in-depth interviews were audio-recorded or not in the appropriate paragraph.
  - We have added a sentence about the recording of the interviews on lines 221-222 in the section about the in-depth interviews.
- Page 10, lines 236-237: Please specify how many interviews were backtranslated
  - On page 10 and in the limitations section at the end of the paper, we clarified that two interviews were backtranslated as a reliability check.
- Pages 10-11: Please report consistent information across authors in the reflexivity section. It is not clear why only EG’s faith is reported and why WB and AAD’s ethnicity, race, or citizenship are not reported.
  - As each author wrote their own reflexivity statement, the information that was presented varies a bit by what the author find most relevant for this research. But we have now made some slight adjustments to make it more consistent across the authors.
- Page 10, line 249: Please clarify how “EG’s positionality as a woman from the Global North affects the research outcomes”
  - To help further elaborate on this statement a sub-clause has been added about her role as an outsider and perceptions of gender equality.
- Page 12, line 302: Please specify which workshop (#1 or #2) is being referred to
  - That statement was in reference to Workshop #2, this has now been changed to say Workshop # 2 instead of the workshop.
- Page 16, line 361: Please clarify the specific observations that made it clear “that not all the faith-leaders agreed with this”
  - In order to clarify this statement we have added a clause that states that based on a debate between the faith-leaders in was clear that they did not all agree.
- Page 25, lines 555-557: Please specify how exactly the gender training in Uganda “impacted overall rates of VAWG”
  - This is important to clarify and the statement was a bit misleading as the data is from focus group discussions with the faith-leaders participating. Thus, we have changed the sentence to say perceptions of VAWG instead.
